# Spatial and genetic dynamics of *Plasmodium falciparum* histidine-rich protein2 in sub-Saharan Africa

**DOI:** 10.1371/journal.pone.0358132

**Published:** 2026-10-07

**Authors:** Kaothar O. Lambe, Favour Adeloye, Jennifer A. Oboh, Halima Abdulsalam, Martin M. Meremikwu, Grant L. Hughes, Eva Heinz, Mary A. Oboh

**Affiliations:** 1 Medical Research Council Unit, The Gambia at The London School of Hygiene and Tropical Medicine, Banjul, Gambia; 2 University of Medical Science, Ondo, Nigeria; 3 Cell Biology and Genetics Department, University of Lagos, Akoka, Nigeria; 4 University of Calabar Teaching Hospital, Calabar, Nigeria; 5 Liverpool School of Tropical Medicine, Liverpool, United Kingdom; 6 University of Strathclyde, Glasgow, United Kingdom; 7 Malaria Genetic Epidemiology Network, NigeriaYaba, Lagos, Nigeria; 8 Biology Department, Lander University, Greenwood, South Carolina, United States of America; Centers for Disease Control and Prevention, UNITED STATES OF AMERICA

## Abstract

**Background:**

Malaria rapid diagnosis tests (mRDTs) detecting *Plasmodium falciparum* histidine-rich protein 2 (*Pfhrp2*) are the most widely adopted diagnostic tool in malaria-endemic areas. However, extensive polymorphisms in *Pfhrp2* could modify *Pfhrp2* epitopes, rendering it undetectable by *Pfhrp2*-based mRDTs. This study investigates the spatial distribution and population dynamics of *Pfhrp2* in sub-Saharan Africa (sSA).

**Method:**

Pfhrp2 sequences (215) were retrieved from public repositories and analysed with MAFFT v7, DnaSP, and various packages in R. We computed genetic diversity metrics, neutrality tests, differentiation, haplotype networks, and phylogenetic analyses.

**Results:**

Varying degrees of insertion-deletion with about 10 polymorphic repeat motif types that varied in frequencies were observed. Repeat types 2, 4 and 7 were the most prevalent types. Baker model prediction with mRDTs monoclonal antibodies indicated the prevalence of highly detected *Pfhrp2* isolates in sSA. Significant diversity was recorded across the sSA region (Hd = 1; θw = 0.09585). The single most dominant haplotype was conserved across countries, and very low differentiation (F_ST_: 0.00–0.04) suggested gene flow between countries.

**Conclusion:**

Our findings imply that the dominant evolutionary pressure in sSA is promoting the parasite's biological fitness and the diversity of *Pfhrp2*.However, the impact of this genetic diversity on diagnostic performance was not assessed as we could not obtain relevant mRDT test data for each country.

## 1. Background

Following the post COVID-19 pandemic, the effect of malaria on the populations at risk in endemic areas has not seen any remarkable decrease, with the World Health Organization (WHO) African region recording the highest malaria cases (89.7%), followed by the Eastern Mediterranean region (15.5%) [1]. In 2023 alone, sub-Saharan African (sSA) region represents 94% of global cases making it the highest contributor of the global malaria cases.

Thus, timely diagnosis would be crucial to initiate effective treatment with antimalarials and prevent progression to severity. As part of the WHO Global Malaria Control Programmes, the T3 initiative “Test Treat Track” has been implemented to control the excessive increase in malaria burden through prompt diagnosis, use of antimalarial, and persistence surveillance [2]. These imply that an appropriate diagnosis is critical before administering any therapy to treat malaria [3].

The WHO has advanced malaria control by incorporating cheaper, easily interpreted, and accessible alternative malaria parasite detection rapid diagnosis test (mRDT) before treatment with artemisinin-based combination therapies [4]. Reports have it that malaria RDT was developed and monitored through pilot studies in several African and South-east Asian countries in the 1990s [5,6]. However, the initial approval and introduction in hospital and commercial laboratory only commenced in the 2000s while wider use for routine diagnosis followed the WHO T3 initiative [6]. Thereafter, several mRDTs were made commercially available and have been designed based on antibodies that detect specific parasite antigens, such as a *Plasmodium* lactate dehydrogenase (LDH), *Plasmodium* aldolase (pALD), and *Plasmodium falciparum* Histidine-rich protein 2/3 (PfHRP2/3) [7]. The commercially available mRDTs targeting HRP2 protein have been designed to detect *P.* *falciparum* only, while those targeting LDH detect both falciparum and non-falciparum malaria [8]. Due to the abundant expression of the *Pfhrp2* during asexual stages, its structural stability, and its multi-epitope nature, mRDTs targeting *Pfhrp2* are the most widely used [9].

Despite these control initiatives, malaria cases have not significantly reduced in the past five years [1]. Genetic variations of *Pfhrp2* have been implicated as a key contributor to the diagnostic challenge encountered in endemic areas since it has been found to result in outright deletion of *Pfhrp2* gene and disorient the gene to form new epitopes that are not detected by mRDT antibody [1]. In the long run, these events may create conditions that select and promote the spread of “diagnostic-evasive” parasite populations, resulting in false-negative results [1]. The evolutionary pressure underlying the emergence and spread of *Pfhrp2* deletions is poorly understood, although, there are pieces of evidence that mRDT-guided treatment of malaria may be the main driver of *Pfhrp2* gene deletions or the key selective pressure of *pfhrp2*-negative parasites. Therefore, survival of the populations is sustained through the transmission and spread of this parasite populations [10,11]. The deletion in *Pfhrp2*, which was initially reported in South America [12] has been detected in several other regions including Asia [13–15] and even Africa [16–18]. This implies that these evasive parasites can become more prevalent in the population, heightening the risk that unnoticed cases may progress to severity, mortality, or even continuous transmission, posing a significant threat to global health [19]. Thus, the WHO guideline recommends continuous baseline surveys of *Pfhrp2/3* deletions in countries where they have been reported as well as their proximate countries [20]. This reveals that deletions directly responsible for false negative result with more than 5% threshold would necessitate a change in mRDT.

The *Pfhrp2* gene is made up of two exons (1 and 2) as well as an intron, and the exon 2 is made up of many amino acid repeats [21,22]. The fact that repeat types 2 and 7, among others, were designated the potential target epitope of monoclonal anti-HRP2 antibodies used in most mRDTs [21,23,24] suggests that variations, especially those involving these repeat types, may contribute to PfHRP2-based mRDT failure [18,24,25]

Given the recent reports on *Pfhrp2/3* gene deletions and genetic variations globally, and their implications especially in the African region, sequence-level surveillance to detect emerging variants that may affect mRDT performance before genetic deletions become prevalent is necessary.

This study investigates the genetic variation of *Pfhrp2* across countries in sub-Saharan Africa. This not only uncovers the geographic dynamics of *Pfhrp2* deletions but also enhance the scope of molecular surveillance, promote data exchange between national initiatives and collaborative approaches to malaria control in Africa.

## 2. Method

### 2.1 Sequence retrieval and inclusion

This study is a secondary bioinformatics and population genetic analysis of publicly available data retrieved from previously published studies. We identified publicly available *Pfhrp2* datasets in published studies obtained from electronic databases such as PubMed (pubmed), NCBI Home Page (ncbi), Sequence Read Archive (sra), Web of Science (web of science), and Scopus (scopus) as well as search engines like Google Scholar (google scholar) and Google using predefined criteria. The key search terms “*Plasmodium*” AND “*falciparum*” AND “Histidine-Rich” AND “Proteins II” OR “PfHRP2” AND “Gene deletion” AND/OR “Genetic Diversity” OR “Genetic Variation” OR “Polymorphism” AND Africa were used. We initially limited our search to observational studies within all WHO African countries; thereafter, we refined the search, including each country in the WHO African region, to identify eligible studies that reported *Pfhrp2* deletion and/or polymorphism. Our search was limited to studies published up to the year 2025.

First, the titles and abstracts of articles identified from our search were reviewed to identify potentially relevant studies. Records that were not original research and unrelated to the study were excluded. Thereafter, relevant articles were selected, and their full texts were retrieved and independently examined. Studies that did not sequence the *Pfhrp2* gene, were conducted outside sSA and that did not clearly state geographical locations of their studies were excluded to ensure that the data is traceable to a location. Following this, three articles were found eligible and retained for further analysis. The accession numbers associated with the *pfhrp2* sequence datasets reported in this studies were obtained to retrieve the datasets from NCBI (ncbi) and GenBank(genbank) databases for downstream *in silico* analysis. The retrieved sequencing data were either full-length or partial regions such as exon 2 of the *Pfhrp2,* and were validated to have met the inclusion criteria.

### 2.2. Sequence alignment and analysis

#### 2.2.1. Sequence alignment.

All retrieved sequences were edited and aligned against *P. falciparum* 3D7 reference obtained from PlasmoDB (PF3D7_0831800) using MAFFT v7 with the --auto, --adjustdirection, and --anysymbol options and the BioEdit software [26] to ensure optimal alignment and orientation. Site-based filtering method was employed to handle missing data such that any alignment position with >20% gaps was removed from the analysis. This threshold is consistent with standard practice in population genetics. For genetic distance calculations (dist.dna with pairwise.deletion = TRUE), missing data were handled by excluding only the specific pairwise comparisons involving gaps, rather than removing entire sequences or sites. The aligned sequences were trimmed at both ends to allow sequences of equal length for downstream analyses.

#### 2.2.2 Deletion and repeat motif analysis.

Analysis of repeat motif was conducted using a custom Python script to identify epitopes of diagnostic relevance, especially Type 1 to Type 12 *Pfhrp2* repeat motifs. Since the data of mRDTs were not available, we employed the Baker model [21] to make a descriptive prediction of mRDT sensitivity based on the diversity of *Pfhrp2*. Thus, isolates were categorized into the following groups based on the product of their type 2 × type 7;Group A, with type 2 x type 7 value of  > 100, followed by Group B(50–100), then, Group I (44–49),and Group C (<43) (Fig 1). The categories distribution was estimated by proportion and the 95% confidence intervals (CIs) of 95% was used to quantify the uncertainty for each proportion. The CIs were computed by the exact binomial method in R using the binom.test function.

**Fig 1:**
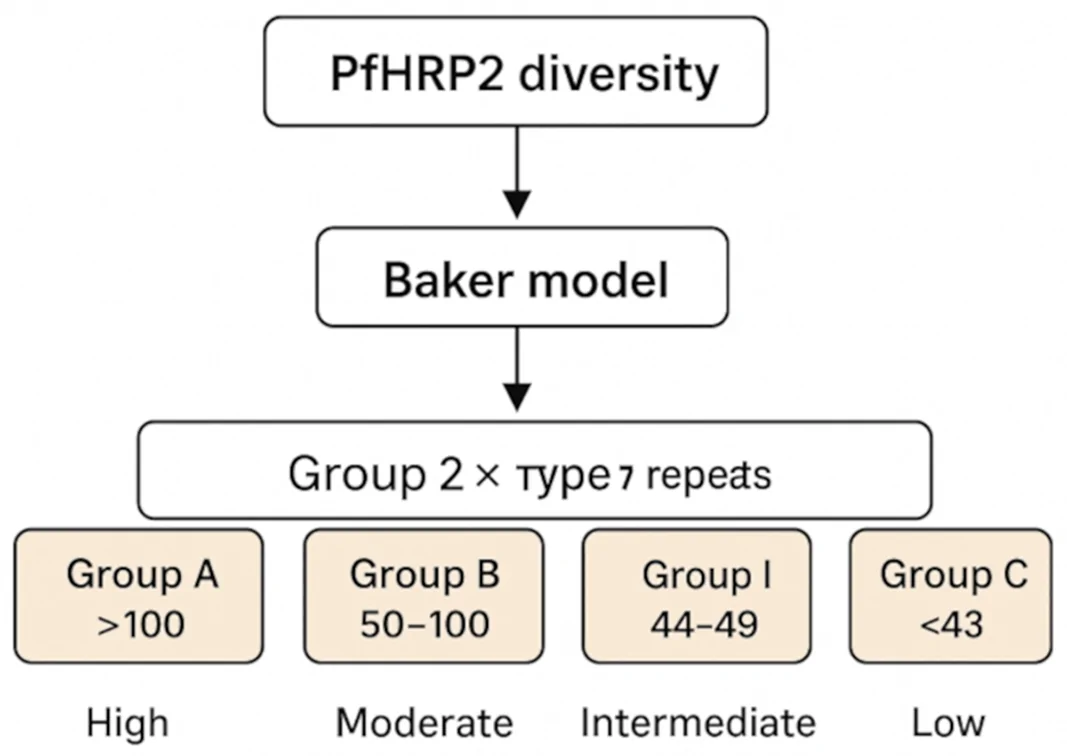
Baker Model for descriptive Prediction of RDT sensitivity. **Legend:** Model made a descriptive prediction of RDT sensitivity based on monoclonal antibodies with the product of motif types 2 and 7.

#### 2.2.3 Evaluation of metrics of genetic diversity.

Diversity metrics, including the number of unique haplotypes and haplotype and nucleotide diversities were estimated for the entire dataset as well as for individual countries. A custom R script built on the ape and pegas packages was employed for these analyses [27,28]. Specifically, the aligned sequences file was fed into hap.div() function from the pegas package to calculate haplotype diversity. However, since the nuc.div() of pegas excludes alignment gaps by default, it was not considered in nucleotide diversity estimation. Alternatively, a custom pairwise function was utilized to compute nucleotide diversity (π), explicitly treating gaps as informative differences. The sequence file was also fed into DnaSP to compute the minimum number of recombination events based on sequence polymorphisms and linkage disequlibrium [24].

### 2.3. Network analysis

To explore the relationships between *Pfhrp2* sequences among various populations in sSA, we constructed a haplotype network using R. The ape package was used to estimate pairwise distances between aligned sequences [28]. Using igraph [29], a range of divergence thresholds (1–5%) was explored to examine the sensitivity of the haplotype. Therefore, a divergence threshold of 2.9% was desirable since it preserved biologically meaningful clusters. This threshold was chosen to capture sequences with low divergence that represent closely related *Pfhrp2* variants, and avoid excessive fragmentation of the network. Based on sensitivity analyses using varying thresholds ranging from 0.01, 0.02, 0.029, 0.03, we observed that 0.029 produced stable clustering without over-fragmentation or excessive merging. Upon plotting a pairwise distance distribution histogram, it was observed that genetic distances were continuous and did not exhibit a clear natural cutoff, so this fixed threshold was applied to define clusters of closely related sequences.For each cluster, we generated a consensus sequence using Biostrings [30]. These sequences were then used to build a second-level network, where edges connected haplotypes with less than 5% pairwise nucleotide difference. In the final visualization, the frequency of each haplotype can be inferred from the node sizes, and pie charts embedded in nodes showed the distribution of samples across various countries.

### 2.4 Genetic Differentiation (F_ST_) of *Pfhrp2*

Pairwise F_ST_ estimates were made to quantify the genetic differentiation among populations that were designed based on countries of origin. Upon reading the aligned sequences file into R, which was fed into the ape package, the sequences were converted into a genind object using adegenet package [31] to ensure gaps were treated as valid alleles rather than missing data. These outputs were then converted into hierfstat format, and pairwise F_ST_ values were computed using Weir and Cockerham's method [32]. To ease visualization and interpretation, the F_ST_ values were used to generate a heat map using pheatmap, and the colour gradient indicates the extent of differentiation among various *Pfhrp2* populations. We also applied a hierarchical based on the Pairwise F_ST_ matrix to identify population structure.

### 2.5 Neutrality tests: Tajima’s D, Fu & Li’s D and F

To estimate any deviation from neutral evolution, we computed Tajima’s D, Fu and Li’s D, and Fu and Li’s F using the PopGenome package [33]. Negative value indicates directional selection (D < 0, F < 0), while a positive value is suggestive of balancing selection [34]. In the analysis, gaps were retained to capture all sequence variation. A general estimate was obtained from the full alignment, and country-specific values were computed from individual country alignments.

### 2.6 Phylogenetic analysis

Evolutionary trend among *Pfhrp2* haplotypes was inferred by a maximum likelihood phylogenetic tree, which was constructed using IQ-TREE2. The haplotype alignment generated from the consensus sequences was used as input, and the best-fitting substitution model was automatically selected with the -m MFP option. Branch support was assessed with 1,000 ultrafast bootstrap replicates (-B 1000). The resulting tree was visualized and annotated in R using the ape, ggtree, and treeio packages [28,35,36]. Tip labels represent country composition of each haplotype, and an alternative tree version was generated where branches and tips were colored according to the dominant country contributing to each haplotype cluster. Custom colour palettes were used for clarity, and tip label sizes were adjusted for legibility.

## 3. Results

### 3.1 Literature search and article eligibility

Of the 446 articles identified after searching through search engines and electronic databases, about 331 duplicates were excluded. The titles and abstracts of the remaining 115 full-text articles were screened. Of these articles, 17 were excluded because they were not original research and 13 excluded because they were unrelated to the study. Of the remaining 85, 55 were also excluded because they had no sequences but genotypes, and 27 excluded because they were either from regions outside Africa were excluded or did not state their study locations. Only 3 articles contributing about 219 sequences of which 4 were excluded because they had poor poor population representation. However, the remaining 215 sequences were included in the final analysis (Fig 2) and they represented 14 African countries (Fig 3).

**Fig 2:**
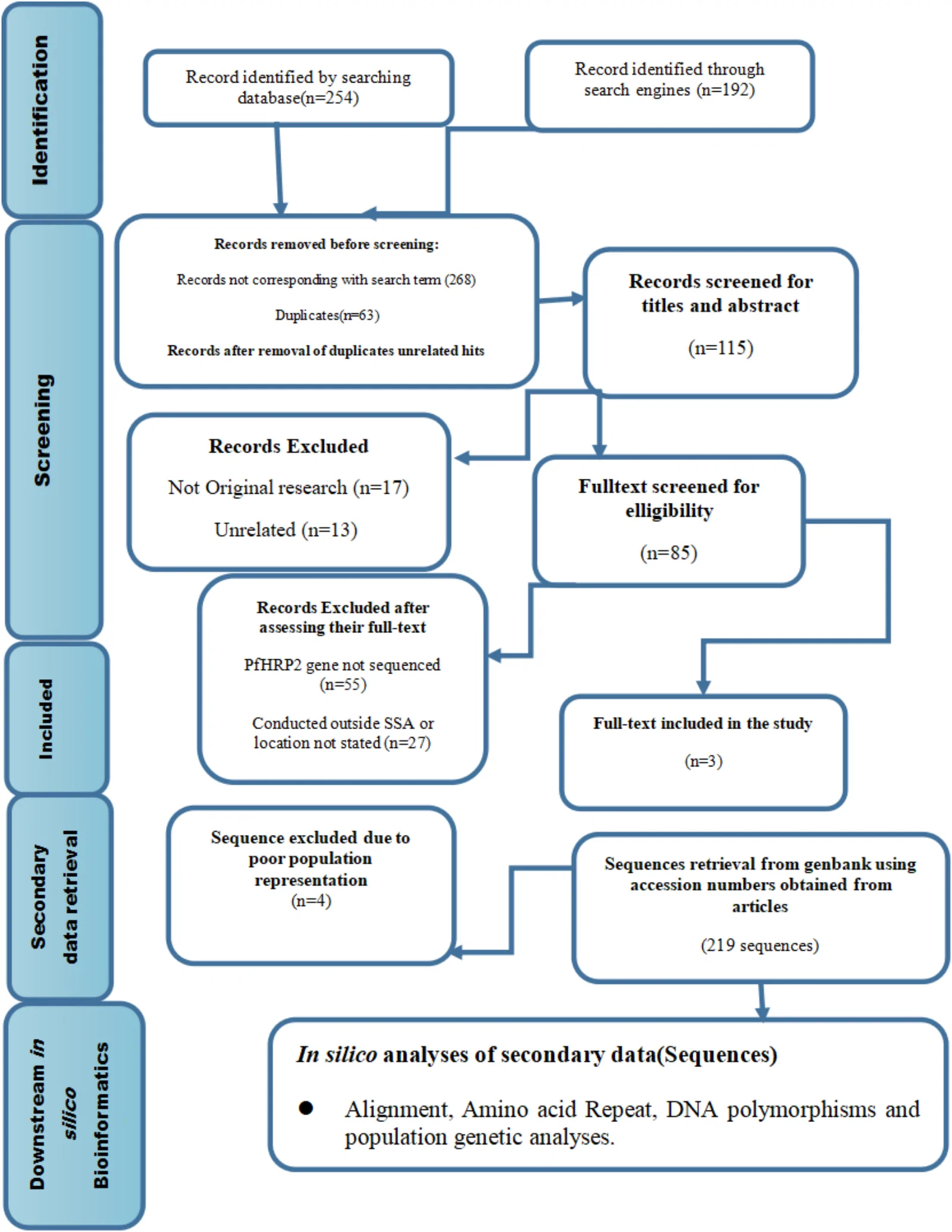
Flowchart of *Pfhrp2* sequence retrieval and inclusion.

**Fig 3:**
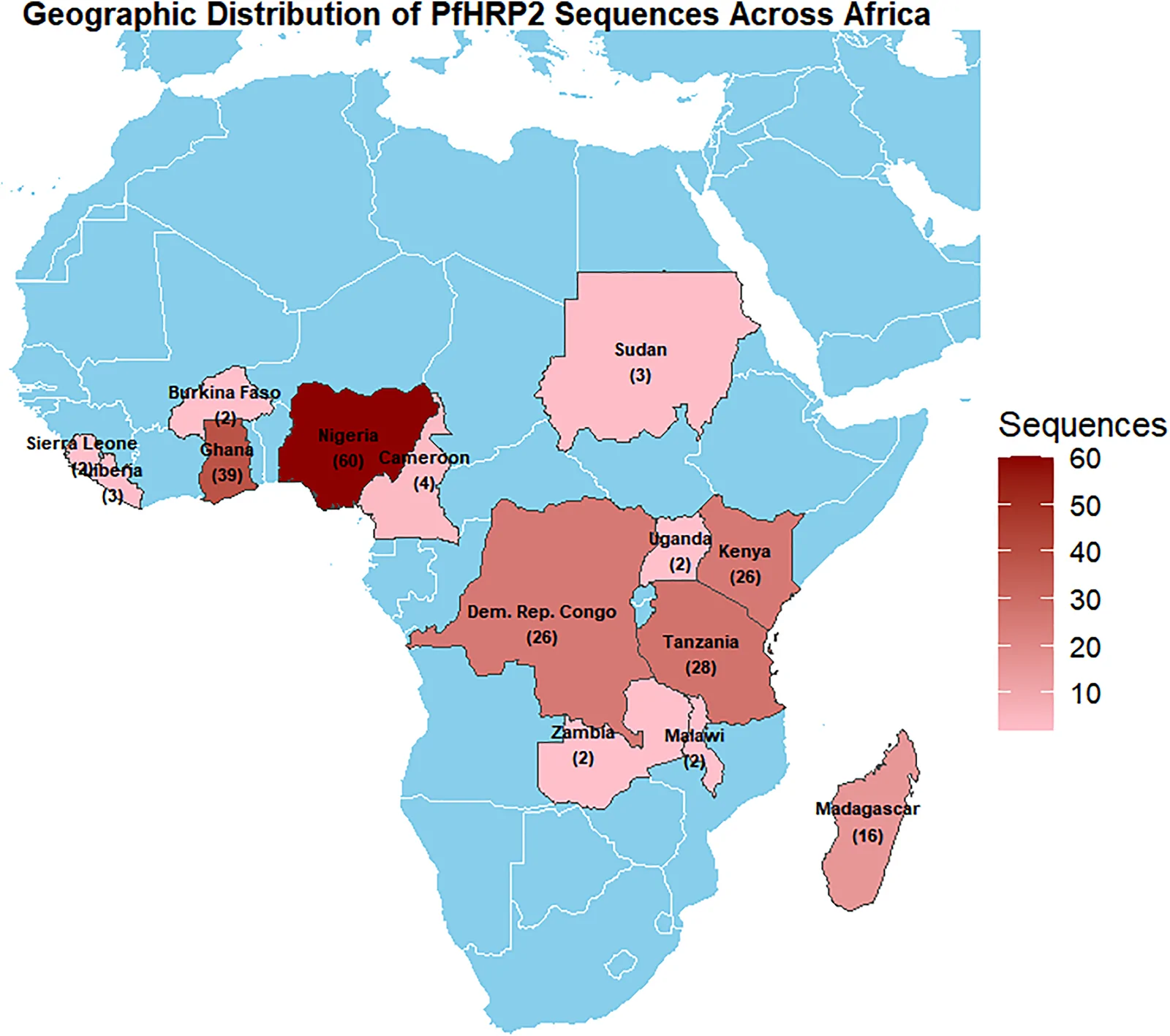
Geographic Distribution of PfHRP2 Sequence Across Africa. **Legend: Density of identified PfHRP2 Sequences across c**ountries are represented by color intensities (light pink = lower sequence count; dark red = higher sequence count). Each country is labeled by its names and number of sequences retrieved in parenthesis.

### 3.2 Retrieved sequence data and alignment

A total of 215 Pfhrp2 exon sequences representing 14 African countries were retrieved from GenBank aligned and saved for downstream analysis (Table 1 and 2),.

**Table 1:** Number of included sequences from each country.

Country	No. of *Pfhrp2* Sequences	Accession Number	References
Burkina Faso	2	AY816237–AY816310	[24]
Cameroon	4	AY816237–AY816310 FJ871160 to FJ871401	[24] [21]
Democratic Republic of Congo	26	FJ871160 to FJ871401	[21]
			
Ghana	39	FJ871160 to FJ871401 AY816237–AY816310 (MT094447−79	[21] [24] [11]
Kenya	26	FJ871160 to FJ871401	[21]
Liberia	3	FJ871160 to FJ871401 AY816237–AY816310	[21] [24]
Madagascar	16	FJ871160 to FJ871401	[21]
Malawi	2	FJ871160 to FJ871401	[21]
Nigeria	60	FJ871160 to FJ871401 AY816237–AY816310	[21] [24]
Sierra Leone	2	FJ871160 to FJ871401	[21]
Sudan	3	AY816237–AY816310	[24]
Tanzania	28	FJ871160 to FJ871401	[21]
Uganda	2	FJ871160 to FJ871401 AY816237–AY816310	[21] [24]
Zambia	2	FJ871160 to FJ871401 AY816237–AY816310	[21] [24]

**Table 2:** Temporal distribution of samples.

Temporal Coverage	Number of samples
**2005-2015**	**182**
**2015-2025**	**33**
**Total**	**215**

### 3.3 Sequence analysis and variation

Based on the 215 sequences from sub-Saharan African countries retrieved from a limited number of studies, the overall haplotype diversity (Hd) between countries was 1, suggesting a high level of *Pfhrp2* haplotype diversity. For within-country analysis, haplotype diversity, of 1 was also recorded for all other countries based on the sequences from selected countries in sub-Saharan Africa. These values reflect high degree of diversity between and within country. However, this finding should be treated with caution because of sampling bias across countries with population (in this case country) sample having small sample size. In countries with minimal number of samples, Hd of 1 may not necessarily be an absolute reflection of underlying diversity but may just connect that the samples are unique and are not a true representation of the *Pfhrp2* diversity in the whole country. We computed the extent of nucleotide diversity within and between countries. The overall nucleotide diversity was 0.0999, suggesting a considerable level of diversity exists between *Pfhrp2* across countries represented by the limited data set based in this analysis. Within each country, the *Pfhrp2* nucleotide diversities varied, with the least computed for Liberia (0.0543), followed by Burkina Faso (0.0556), and Zambia (0.0680). However, the highest nucleotide diversity (0.0827–0.16495) based on the limited dataset computed for most countries include Malawi, Kenya, Sudan, Tanzania, Uganda, Ghana Nigeria, Madagascar, Democratic Republic of Congo (DRC), Sierra Leone, and Cameroon (Table 3).

**Table 3:** Metrics of diversity and neutrality tests.

Country	N	HD	ND	TD	Fu & Li’F	Fu & Li’D	Rm
Burkina Faso	2	1	0.0556	NA	NA	NA	NA
Cameroon	4	1	0.1449	−9.572108^e^	2.44524^a^	2.30909^a^	2
DRC	26	1	0.1144	−3.604369^e^	1.31089^a^	1.82043	17
Ghana	39	1	0.0910	−3.245164^e^	−0.55019^a^	−0.53471^a^	35
Kenya	26	1	0.0844	−3.343126^e^	0.02793^a^	−0.19005^a^	19
Liberia	3	1	0.0543	NA	NA	NA	0
Madagascar	16	1	0.0988	−3.706252^e^	−1.55854^a^	−1.91077^a^	18
Malawi	2	1	0.0827	NA	NA	NA	NA
Nigeria	60	1	0.0925	−3.141449^e^	−0.15993^a^	−0.2904^a^	17
Sierra Leone	2	1	0.1236	NA	NA	NA	NA
Sudan	3	1	0.0863	-NA	1.31089^a^	1.82043^a^	0
Tanzania	28	1	0.0890	−3.353359^e^	−1.08793^a^	−1.26823^a^	20
Uganda	2	1	0.0908	NA	−0.55019^a^	−0.53471^a^	0
Zambia	2	1	0.0680	NA	NA	NA	NA
Overall	206	1	0.0999	−2.890632^e^	−2.136416^a^	−2.220828^a^	9

**NB**: All sites considered are parsimony informative sites, no singleton was included.

a-P > 0.10 e-P < 0.005

N: Number of haplotypes; HD: Haplotype Diversity; ND: Nucleotide Diversity

TD: Tajima’s D; Rm: Minimum number of recombination event

### 3.4. Neutrality test and signatures of *Pfhrp2* selection

To estimate whether *Pfhrp2* deviates from the neutrality immune selection model, we computed a neutrality test. The overall Tajima’s D value is indicative of directional selection (−2.890632), which was statistically significant at (P < 0.005). To confirm this, we equally conducted the Fu and Li’s D (−2.136416) and F tests (−2.220828) which were not statistically significant at (P > 0.10). The neutrality metrics varied between countries, reflecting local variation in *Pfhrp2* selection pattern. Equally, the Tajima’s D values computed for all countries Cameroon, DRC, Ghana, Kenya, Nigeria, Madagascar, and Tanzania (−9.572108, −3.604369, −3.245164, −3.343126, −3.706252, −3.141449& −3.353359) respectively were suggestive of directional selection. The deviations from the neutrality model estimated by Tajima’s D were statistically significant while the FU and Li’F and D could not confirm these observations as the recorded values were not statistically significant (Table 3).

### 3.5 Variation and repeat Motif Analysis

#### 3.5.1 And amino acid repeat frequency and variation.

There were significant diversity within the exon 3 of *Pfhrp2.* This was possibly due to variations in the numbers and arrangement of each repeat motif in this exon 2 which result in differences in the length and composition of the sequences (Table 4). A total of ten distinct amino acid repeat types were identified across the isolates. Only three repeat types(2,4,7) occurred at the highest frequency and were present in all isolates (100%). However, We observed a semi-conserved motif of repeat in the exon 2 of the sequences, consisting of repeat types 2, 3, 5, 7, 8, 2, and 7 in about 162 of 219 sequenced isolates (74%), as well as a partial amino acid motif composed of 7,8,2,7 at 99.5%. In addition, types 6, 8, and 9 were present at 99.6%, while Types 1, 3, and 10 were present at 97.8%, 91.5% and 83.3% respectively. while the least frequent was Type 5 (77%) (Table 4). The number of repeats per isolate slightly varied within each country but not between countries. Uniquely, Type 3 was absent in all isolates in Burkina Faso, and Type 10 was only present in 50% of the isolates from DRC.

**Table 4:** Repeat motif type identified.

Code	Repeat sequences	Frequency	Percentage
Type 1 motif	AHHAHHVAD	214	97.7
Type 2 motif	AHHAHHAAD	219	100
Type 3 motif	AHHAHHAAY	202	92.2
Type 4 motif	AHH	219	100
Type 5 motif	AHHAHHASD	168	77
Type 6 motif	AHHATD	218	99.6
Type 7 motif	AHHAAD	219	100
Type 8 motif	AHHAAY	218	99.6
Type 9 motif	AAY	218	99.6
Type 10 motif	AHHAAAHHATD	193	83.3

#### 3.5.2 Statistical prediction of RDT performance by the baker model.

We made a descriptive prediction of the sensitivity of mRDT based on the *Pfhpr2 diversity* using the Baker’s model. The result indicated that group A, ‘the strongly detected’ were the most prevalent (97.7%, 95% CI: 94.7–99.2%) which are of high RDT sensitivity, followed by group B occurring at (1.9%, 95% CI: 0.5–4.7%) ‘usually detected’ while group C, which are designated as ‘may escape detection’ was the least prevalent is (0.5%, 95% CI: 0.01–2.6%) (Fig 4).

**Fig 4:**
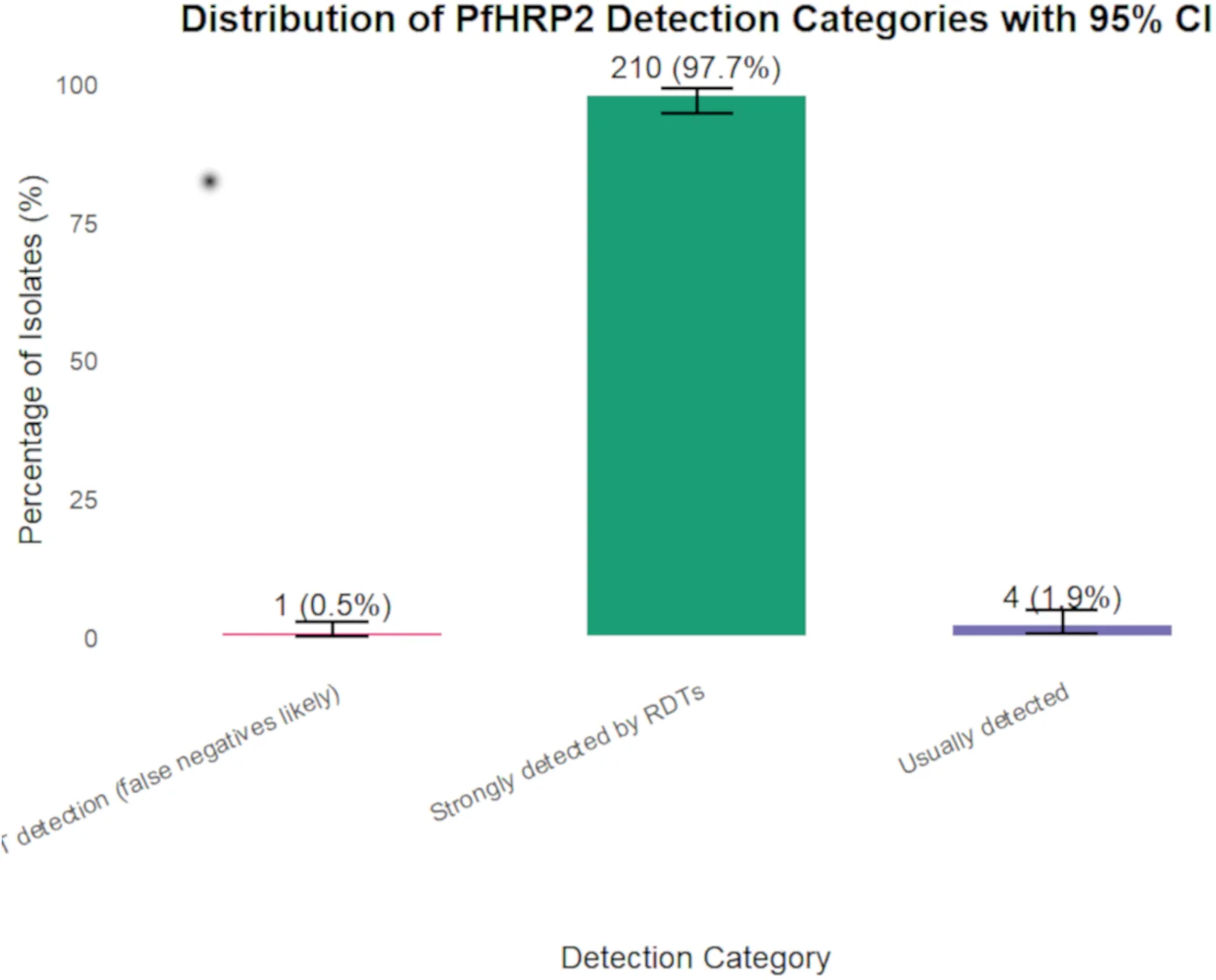
Prevalence of Baker Model categorization of *Pfhrp2*-based RDT Sensitivity across sub-Saharan Africa at 95% CI. **Legend**: The highly sensitive group (GroupA) was the most prevalent in sSA, others were scarce. A confidence interval of 95% was adopted to quantify the uncertainty for each proportion.

### 3.6 Haplotype network

We constructed a haplotype network to explore the evolutionary relationship between haplotypes of *Pfhrp2* from different countries represented by sequences in our study (Fig 5). From the total haplotypes inferred in sSA based on our dataset, the selected threshold clustered sequences with less than 30% nucleotide differences are more evolutionarily related than others. Thus, the coalesce resulted in 38 haplotypes consistent with significant diversity metrics recorded for most countries. Each node representing a haplotype was coloured differently with respect to its locality, as depicted in the legend. The largest node (hap 1)is the most dominant haplotype and represents isolates from multiple countries, suggesting it is a largely shared or conserved haplotype. In addition, hap 3 was shared among Madagascar, Nigeria, Ghana, Democratic Republic of Congo, Sudan, Tanzania and, Kenya while hap 11 was shared among Madagascar, Ghana and Nigeria. These patterns reflect gene flow between these countries.

**Fig 5:**
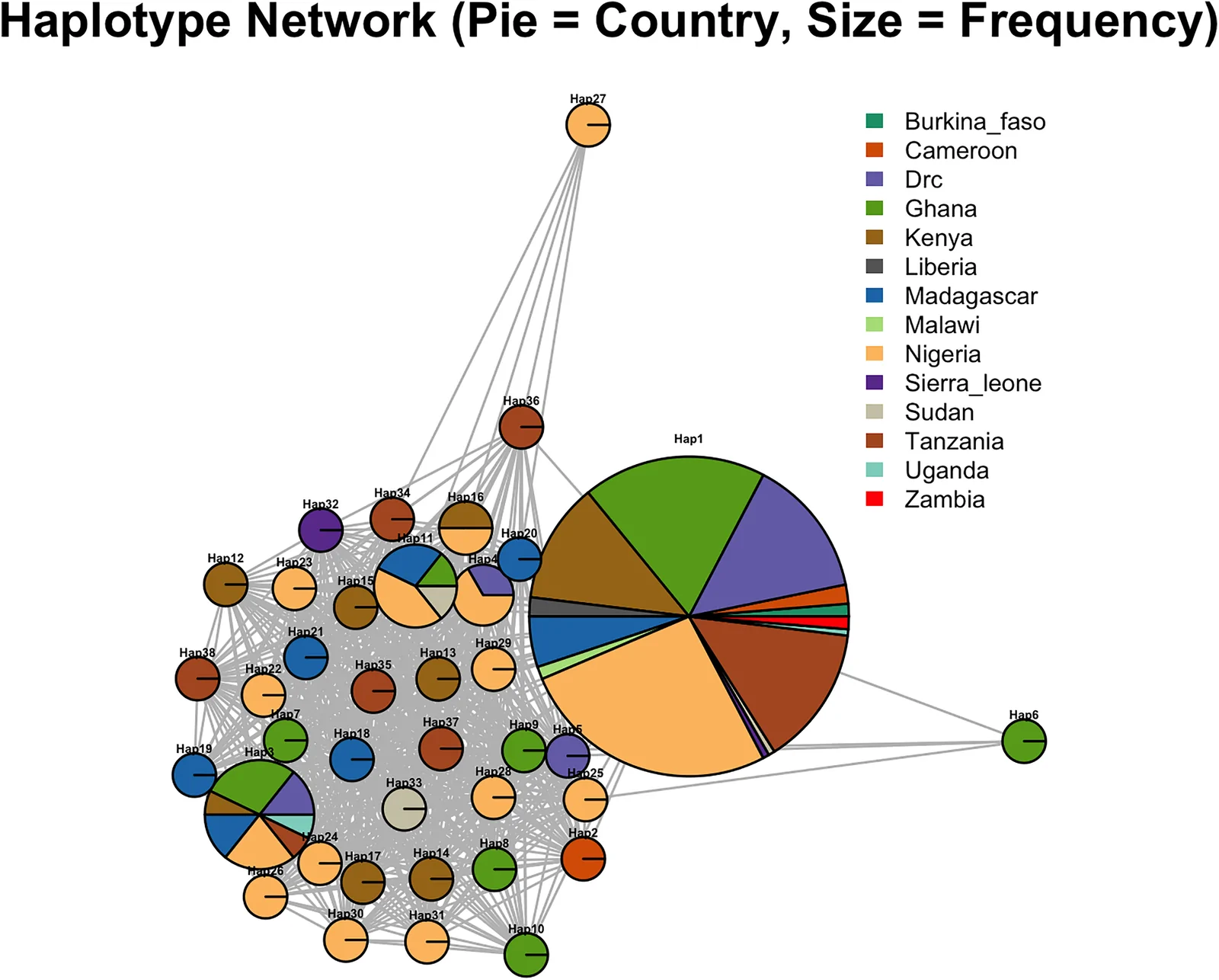
a. Distance Matrix represented plotted with a histogram b. Representative isolates of sub-Saharan African *Pfhrp2* haplotype network. **Legend:** Isolates representing each countries have been clustered based 30% similarities, hence there were 38 haplotypes, each represented by nodes and coloured by country its represent. Some nodes are shared into parts and multi-coloured implying that they were shared by multiple countries. In particular, the large node at the centre was shared across several countries. The edges connect haplotypes differing by one or several mutations.

Interestingly, most countries, including,Ghana, Kenya, Nigeria, Sierra Leone, Tanzania, and Madagascar, possessed several singleton haplotypes that were not grouped by country. However, some smaller haplotypes from Madagascar, DRC, and, Nigeria were connected around the large one, reflecting evolutionary relatedness to the large one. In addition, a few ones from Ghana, Nigeria, and Tanzania were farther apart from others and one another indicating they do not share recent ancestors or are newly emerging variants(Fig 5).

### 3.7 Gene flow and genetic differentiation

We computed pairwise F_ST_ to estimate differentiation among *Pfhrp2* populations in sSA and visualized them with a heat map. Overall, the heat map reflected slight differentiation among *Pfhrp2* populations in sSA. Equally, the applied colour gradient reflected the extent of differentiation; cells with lighter shades of a colour showed no differentiation at the intersects, the darker shades indicated points of slight genetic differentiation (Fig 6). These observations reflected extensive gene flow, making the *Pfhrp2* diversity similar in almost all populations.

**Fig 6:**
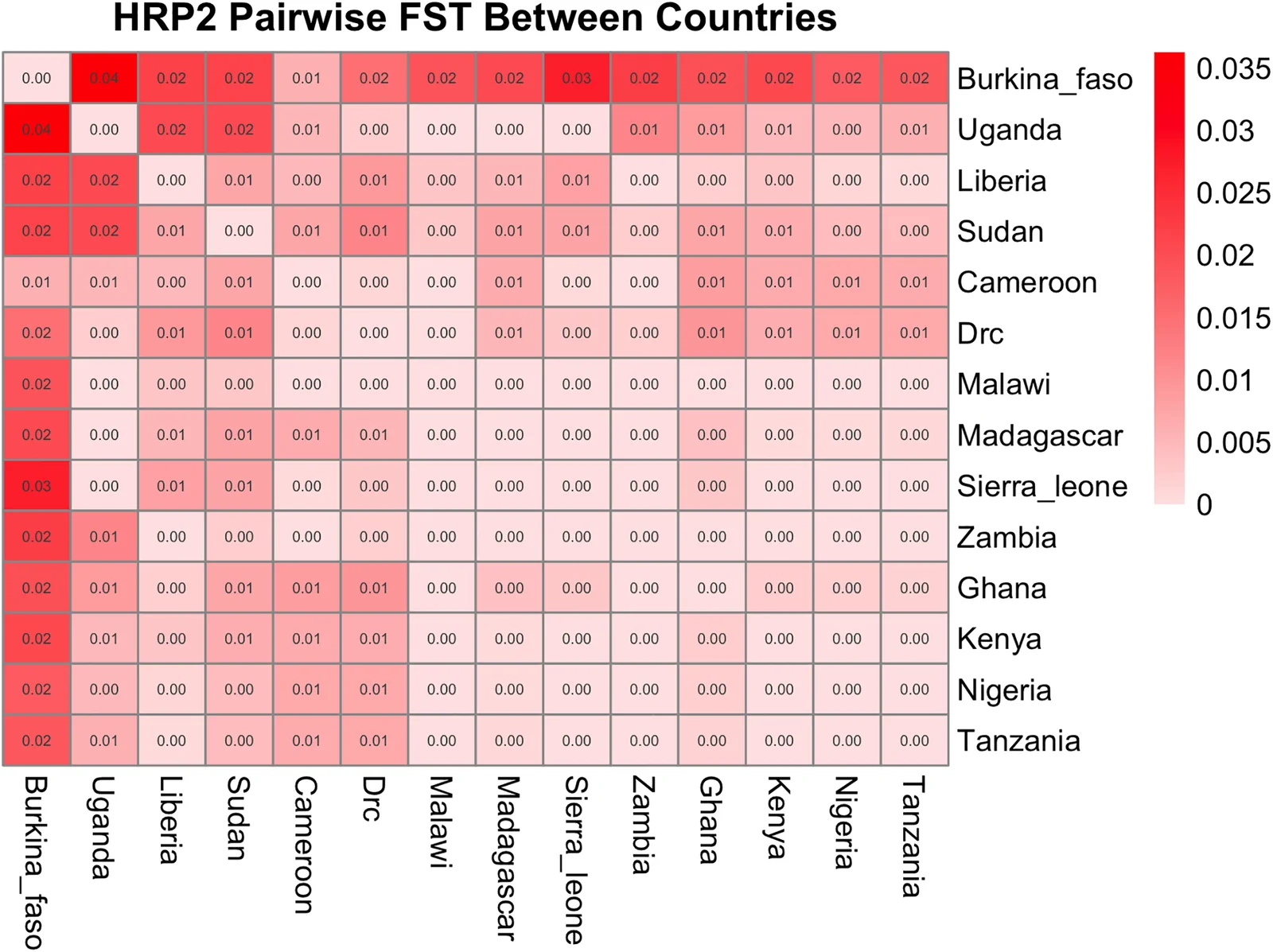
Population Differentiation of Pfhrp2 among isolates from various populations in sSA. **Legend**: A colour gradient was used on the heatmap to indicate the extent of differentiation inferred by pairwise F_ST_ between populations. The differentiation increases with increasing colour intensities, cells with lighter shades of a colour showed no differentiation at the intersects, while the darker shades indicated points of slight genetic differentiation.

### 3.8 Phylogenetic analysis

We predicted evolutionary relationships between isolates from various countries within sSA by a phylogenetic tree and explored the possible clustering by lineage. The phylogenetic tree (Fig 7) shows that all *Pfhrp2* isolates originated from a common ancestral lineage, which branches into three major sister clades, indicating the presence of three primary evolutionary lineages of *Pfhrp2,* which are not exclusive to a country. Haplotypes such as Hap 1, 3, and 11 consist of isolates from multiple countries. Uniquely, Hap 1 was the only haplotype found in the third and lowest clade and consists of isolates from all countries, suggesting that it may be an ancestral lineage that is conserved despite the overall diversity. Its presence in several countries may also imply population expansion and has been widely spread in Africa by extensive gene flow. However, each of the other two major clades branches into several sub-clades that are even more genetically related. The first and upper clade (clade one) consists of fewer isolates and forms two main sub-clades that even form smaller branches. The middle second branches into two major sub-clades; the upper sub-clade bearing a haplotype (hap 29) restricted to only Nigeria, the second lower sub-clade which even branches more sub-clades that were consist majorly of non-country-specific haplotypes due the displayed extensive geographic admixing among haplotypes from different African countries, reflecting extensive gene flow. For instance, Hap 36 from Tanzania was more genetically related, sharing a common recent ancestor with 32, 22, and 13 from Sierra Leone, Nigeria, and Kenya, respectively, than Hap 38 from the same Tanzania.

**Fig 7:**
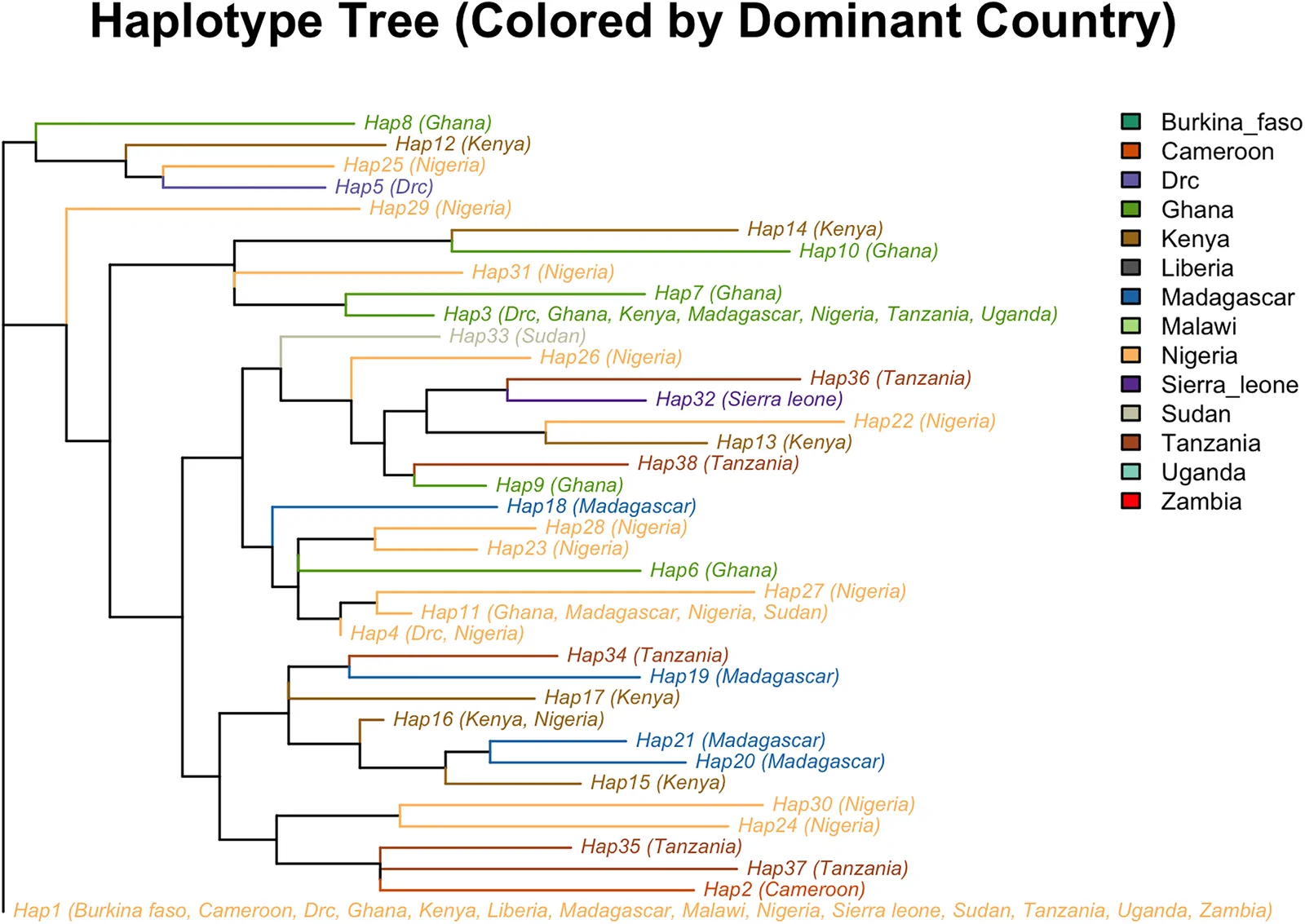
Phylogenetic relationships among *Pfhrp2* from various sub-Saharan African Countries. **Legend:** Phylogenetic tree of *pfhrp2* haplotypes identified among isolates of P. falciparum across African populations. Three major sister clades were observed indicating the presence of three primary evolutionary lineages of *Pfhrp2,* which are not exclusive to a country. Each branch at the extreme represents a unique haplotype and the labels are colour-coded according to the country contributing the majority of sequences within that haplotype, as shown on the picture legend. The lengths of the each branch reflects the extent of genetic diversity among haplotypes.

Despite overall geographic mixing in this second clade, the lower sub-division of sub-clade 2 notably exhibited clusters that were dominated by isolates from the same country. For instance, Haps 30 and 34 from Nigeria tend to cluster together, same as Haps 20 and 21 from Madagascar, while Haps 31 and 37 of Tanzania also cluster together. This pattern reflects recent local parasite transmission and diversification despite overall continental admixing (Fig 7).

## 4. Discussion

Since its recommendation by the WHO, malaria RDT (mRDT) has been adopted as a reliable and cost-effective diagnostic tool for malaria in settings where quality-assured microscopy is not feasible, especially in resource-constrained settings such as sSA. Here, malaria cases and mortality are highly prevalent, but microscopy cannot be easily accessible. Some of these mRDTs are *PfHRP2*-based, implying that they target the HRP2 protein released by the parasite into peripheral circulation. Unfortunately, there are concerning reports on the widespread deletion as well as extensive polymorphism in *Pfhrp2* coding for the HRP2 antigen, as these threaten the diagnostic performance of mRDTs [12,37] and, on a broader level, obstruct malaria control. This study employs population genetic analyses to investigate the genetic diversity in *Pfhrp2* from *P. falciparum* field isolates in sSA between 2005–2025.

Deletions in the *Pfhrp2* gene have been reported in multiple locations at varying prevalence, including 0.016% in Senegal, 2% in Mali, 36% in Ghana, 14% in Surinam, 23% in Rwanda, 41% in Guyana, and Peru [12,18,38–41] suggesting an emerging threat to HRP2-based diagnosis in sSA. Remarkably, we did not find true deletions that may pose significant threat to diagnostic performance, rather we found considerable number of polymorphisms in amino acid repeats contributing to the overall variations in the gene. Interestingly, there are a few evidence that variation in *Pfhrp2* repeat numbers could affect the diagnostic performance of HRP2-based mRDTs [18,24,25]. Analyses of the *Pfhrp2* gene revealed the presence of 10 of the known repeat types [24]. Our analysis revealed that repeat types 2,4,6,7, and 8 were among the most frequent repeat motifs as they were present in over 99% of the isolates analysed in this study. This is consistent with the observations of many experimental studies at the country-level in Africa, America, and Asia [21,22,42–44], which mostly observed 2,6,7, and 8 in all isolates. Type 11 was totally absent in this study, consistent with the observations from some previous studies of isolates from Uganda, and Madagascar that lacked this repeat type [18,25,44]. However, our observation slightly contradicts former studies as type 9 was totally absent in isolates analyzed in their work, while it was present in over 99% of all isolates included in the current meta-analysis. Almost all isolates from countries included in this study began with repeat type 1; this is consistent with the findings of most studies that found that all isolates began with type 1 repeats [14,24,25,44]. Conversely, five isolates did not begin with type 1; this observation aligns with previous reports from Senegal and DRC [18,21] that found three isolates each not beginning with type 1. Interestingly, about 2 out of the 5 isolates that did not begin with repeat type 1 in our analysis were from DRC while the remaining three were distributed among Ghana, Nigeria and Tanzania.

In addition, the high level of genetic diversity among *Pfhrp2* isolates from sSA, indicated by both nucleotide and haplotype diversities, is comparable with that recorded for *Pfhrp2* gene by other previous studies in African countries, including Ghana, Senegal, Madagascar, Ethiopia and Kenya [3,18,22,25,45]. The significant diversity in *Pfhrp2* is typical of genes at the telomeric and sub-telomeric regions of *Plasmodium* species, since they are highly susceptible to variation due to high recombination events [21,46]. Typically, in malaria endemic areas, transmission is intense increasing the likelihood of polyclonal *Plasmodium* infection that results in extensive genetic recombination in the mosquito vectors [47]. Consequently, the minimum numbers of recombination events computed across many countries were commensurate with the observed high diversities. Similarly, the host immunity may select for immune-relevant variants thus contributing to the overall *Pfhrp2* diversity in sSA [21]. Also, the high level of diversity may have resulted from selective pressure that arises from sustained use of HRP2-based mRDTs as this has been predicted to promote the survival of evasive parasites in certain context [48]. However, the first confirmed case of *Pfhrp2* deletion in South America was prior to the introduction of mRDTs suggesting that the evasive may possess biological fitness such that increased mutation frequency is maintained even without diagnostic pressure [12,49]. Further, neutrality test detected signatures of balancing selection in a few locations including Madagascar, Cameroon, and Tazania, suggesting a simultaneous effect of recombination and immune-mediated variation in haplotypes for the parasite to evade antibody detection [50–52]. In addition to the pattern of variation in repeat-number observed in all countries reflecting that specific repeat motif types, including 2, 4, and 7 were being favoured, the overall neutrality test is suggestive of directional selection acting across countries. Evidently, the HRP2 protein has been partially implicated in the pathogenesis of the parasite to facilitate heme detoxification, thus ensuring the parasite’s survival [4,53,54], but a larger function of heme detoxification has been attributed to haem detoxification protein (HDP) more recently [55]. Thus, isolates lacking HRP were found to have survived [56]. in this study, the repeat motif types with high frequency were being selected, and their frequency expanded probably because they enhance robust protein expression in manners that do not put the parasite at any fitness cost. This implies that isolates with high frequency repeats will likely be more biologically fit, ensuring survival and continuous transmission. However, such isolates are easily detectable as the mRDT may be highly sensitive to their intact HRP2. This finding is consistent with the predicted mRDT sensitivity in this study based on HRP2 antibodies according to Baker model [21]. Notably, the applicability of this model for newer mRDT performance maybe limited due to changes configuration of antibody in newer diagnostic devices, but we only adopted to make as a descriptive estimate of the expected diagnostic performance and not definitive prediction. Further, with respect to our limited datasets, the observed diversification may not be locally restricted since the computed F_ST_ indicated no remarkable genetic differentiation at the *Pfhrp2* locus in sSA. This observation may be consistent with extensive gene flow facilitated by geographic proximity and human migration [57,58]

Furthermore, the largest haplotype, which consist of isolates from several nations with few derived ones attached to it reflecting similarity, may be conserved and has only accrued a small number of mutations throughout time, presumably due to country-specific selective pressure. As a result, despite indicated minimal population differentiation, we found many distinct haplotypes in numerous countries, which is in line with the wide *Pfhrp2* diversities recorded. Similar results have been documented in Ghana, where, despite the claimed low differentiation, multiple distinct *P. falciparum* haplotypes from two distinct locations were discovered. However, those unique haplotypes have some degree of homology with specific shared haplotypes, which suggest that they might have evolved from the shared haplotype through mutations under region-specific selective pressures [49]. Accordingly, our phylogenetic tree did not show clades that were unique to a particular country. This could be because high genetic recombination regularly leads to the exchange of genetic materials between isolates of different origins, preventing the formation of country-specific lineages by unique haplotypes. Consistently, the low F_ST_ as well as inferred low differentiation may indicate extensive gene flow due to human migration between countries, which results in genetic mixing between isolates of various backgrounds [59]. However, while this observation may be consistent with extensive gene flow, there is a need to interpret this cautiously. This is because the uneven sample size across countries may limit the reliability of F_ST_, thereby potentially masking the population structure. Consequently, we can only infer that the low F_ST_ may be indicative of extensive gene flow and not definitely stating that the gene flow in the region is extensive.

Overall, while previous observational studies in a few sub-Saharan African countries recorded deletions in *Pfhrp2* gene at low frequencies, reflecting emerging diagnostic escape, our findings revealed a high level of diversity, suggesting ongoing evolution rather than outright deletion among *Pfhrp2* in sSA. Equally, our findings suggest that from the representative data included in the study, the dominant evolutionary pressures in this population may promote biological fitness and genetic diversity of *Pfhrp2,* but their impact on diagnostic performance cannot be definitely inferred from this present study.

Given the limitations of this study, there is therefore a need for more robust observational studies with large and even sample size across the continent to give more comprehensive picture of the genetic diversity and population genetic patterns of the *Pfhrp2* gene across the continent. More specifically, it would be more relevant to strengthen surveillance of *Pfhrp2* gene diversity and deletions, their evolutionary drivers and potential impact on diagnostic performance in WHO regions such as sSA with of high concern of HRP loss.
